# The TrialsTracker: Automated ongoing monitoring of failure to share clinical trial results by all major companies and research institutions

**DOI:** 10.12688/f1000research.10010.1

**Published:** 2016-11-03

**Authors:** Anna Powell-Smith, Ben Goldacre

**Affiliations:** 1 Evidence-Based Medicine Data Lab, Centre for Evidence-Based Medicine, Nuffield Department of Primary Health Care Sciences, University of Oxford, Oxford, UK

**Keywords:** Publication bias, transparency, audit

## Abstract

*Background*: Failure to publish trial results is a prevalent ethical breach with a negative impact on patient care. Audit is an important tool for quality improvement. We set out to produce an online resource that automatically identifies the sponsors with the best and worst record for failing to share trial results. *Methods:* A tool was produced that identifies all completed trials from clinicaltrials.gov, searches for results in the clinicaltrials.gov registry and on PubMed, and presents summary statistics for each sponsor online. *Results*: The TrialsTracker tool is now available. Results are consistent with previous publication bias cohort studies using manual searches. The prevalence of missing studies is presented for various classes of sponsor. All code and data is shared. *Discussion:* We have designed, built, and launched an easily accessible online service, the TrialsTracker, that identifies sponsors who have failed in their duty to make results of clinical trials available, and which can be maintained at low cost. Sponsors who wish to improve their performance metrics in this tool can do so by publishing the results of their trials.

## Introduction

The results of clinical trials are used to make informed choices with patients about medical treatments. However, there is extensive and longstanding evidence that the results of clinical trials are routinely withheld from doctors, researchers, and patients. A current systematic review of all cohort studies following up registered trials, or trials with ethical approval, shows that approximately half fail to publish their results ^1^. Evidence from an earlier review shows that studies with “negative” or non-significant results are twice as likely to be left unpublished ^2^. Legislation, such as FDA Amendment Act 2007 ( http://www.fda.gov/RegulatoryInformation/Legislation/SignificantAmendmentstotheFDCAct/FoodandDrugAdministrationAmendmentsActof2007/default.htm), which requires trials to post summary results on clinicaltrials.gov within 12 months of completion, have been widely ignored, with a compliance rate of one in five ^3, 4^. The FDA is entitled to impose fines of $10,000 a day on those breaching this law, but has never yet done so ^5, 6^. This public health problem has also been the subject of extensive campaigning. For example, the AllTrials campaign is currently supported by 89,000 individuals and 700 organisations, including major funders, professional bodies, patient organisations and government bodies ( http://www.alltrials.net/).

Previous work suggests that some sponsors, companies, funders, and research sites may perform better than others ^5, 7^. In any sector, audit of the best and worst performers can be used to improve performance, allowing those with a poor performance to learn from those doing better. To be effective, however, audit should be repeated, and ideally ongoing ^8^.

All work on publication bias to date relies on a single sweep of labour-intensive manual searches ^9^, ^10^, or a single attempt to automatically match registry entries to published papers using registry identification number ^11^. Manual matching comes at high cost and does not give ongoing feedback. We therefore set out to: develop an online tool that automatically identifies trials with unreported results; present and rank the prevalence of publication failure, broken down by sponsor; and maintain the service, updating the data automatically, so that companies and research institutes are motivated to improve their performance.

## Methods

The methods used by the online tool are as follows. Raw structured data on all studies in clinicaltrials.gov are downloaded in XML format. Studies are kept if they: have a study type “interventional” (excluding observational studies); have a “status” of “completed”; have a completion date more than 24 months ago, and after Jan 1 2006; are phase 2, 3, 4, or “n/a” (generally a device or behavioural intervention); no application to delay results posting has been filed (ascertained from the *firstreceived_results_disposition_date* tag); are conducted by a sponsor who has sponsored more than 30 trials (to exclude trials conducted by minor sponsors and make the ranking in the tool more informative).

Results are then sought for all included studies, using two methods. First the tool checks for structured results posted directly in clinicaltrials.gov, ascertained by the presence of the *firstreceived_results_date* tag. Secondly, the tool searches for the *nct_id* (registry ID number) of the trial in PubMed’s *Secondary Source ID* field. Since 2005, all trials with a registry ID in the body of the journal article text should have that ID replicated in this field ( https://www.nlm.nih.gov/bsd/policy/clin_trials.html). However, since in our experience approximately 1.5% of PubMed records include a valid *nct_id* list in the abstract, but not the *Secondary Source ID* field, our tool additionally searches for this ID in the title or abstract text. We exclude results published before the completion date of the trial, or results that have the words “study protocol” in the title.

A final filter is then applied, with the aim of excluding publications reporting protocols or additional analysis and commentary, rather than trial results; after experimenting with the standard validated PubMed “therapy” filters (both broad and narrow) and a rudimentary search for “study protocol”, the former was used. A comparison of the three methods is reported in the accompanying iPython notebook [ https://github.com/ebmdatalab/trialstracker] ^12^.

Accepting that an automated tool cannot produce results with the accuracy of a manual search, we also performed some rudimentary checks of the output of the automated search against existing manual search cohorts. The overall prevalence of unreported studies found by the tool was compared against three previous studies on publication bias. In addition, disparities on individual studies found to be unreported by the tool were compared against the underlying data from a recent publication bias cohort study conducted using clinicaltrials.gov data. 

The output data is then shared through an interactive website at https://trialstracker.ebmdatalab.net allowing users to rank sponsors by number of trials missing, number of trials conducted, and proportion of trials missing. Users can click on a sponsor name to examine the number and proportion of trials completed and reported from each year for that sponsor. The site URL changes as users focus on each organisation’s performance, so that users can easily share insights into the performance of an individual company or institution. By default sponsors are sorted by the highest number of unreported trials, rather than the highest proportion, in order to initially focus on larger and more well-known organisations. The site is designed responsively to be usable on mobile, tablet or desktop devices.

For transparency and replication, all code for the tool, with comments and all data sources, is available as an iPython notebook ^12^. All software is shared as open source, under the MIT license. A full CSV is shared containing all data, including all studies before our filters are applied, allowing others to conduct additional analyses or sensitivity analyses with different filtering methods.

## Results

**Figure 1.**
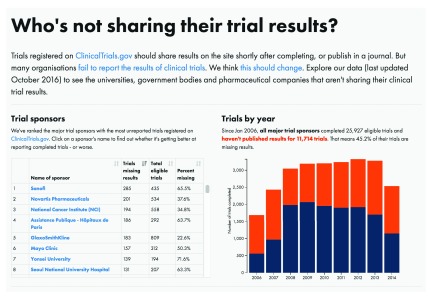
Screenshot, all trials. https://trialstracker.ebmdatalab.net/.

**Figure 2.**
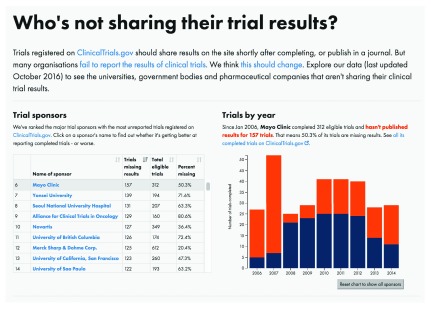
Screenshot, all trials by Mayo Clinic. https://trialstracker.ebmdatalab.net/#mayo-clinic.

The TrialsTracker tool was successfully built and is now running online at https://trialstracker.ebmdatalab.net. Sample screenshots are presented in Figure 1 and Figure 2.

Since Jan 2006, trial sponsors included in our dataset have completed 25,927 eligible trials, of which 11,714 (45.2%) have failed to make results available. Table 1 to Table 4 report the sponsors with the top five highest number of unreported trials, the highest number of eligible trials, the highest proportion of unreported trials, and the lowest proportion of unreported trials. In total, 2390/8799 (27.2%) trials with sponsors classed as “industry” were identified as unreported; 122/470 (26.0%) trials with sponsors classed as “US Fed” were identified as unreported; 361/996 (36.2%) trials with sponsors classed as “NIH” were identified as unreported; 8841/15662 (56.4%) trials with sponsors classed as “other” were identified as unreported. We find that 8.7 million patients were enrolled in trials that are identified as unreported.

**Table 1.** Top five sponsors with the highest number of missing results. TrialsTracker, 20/10/2016.

Name of trial sponsor	Trials missing results	Total eligible trials	Percent missing
Sanofi	285	435	66%
Novartis Pharmaceuticals	201	534	38%
National Cancer Institute (NCI)	194	558	35%
Assistance Publique - Hôpitaux de Paris	186	292	64%
GlaxoSmithKline	183	809	23%

**Table 2.** Top five sponsors with the highest number of eligible trials. TrialsTracker, 20/10/2016.

Name of trial sponsor	Trials missing results	Total eligible trials	Percent missing
GlaxoSmithKline	183	809	23%
Merck Sharp & Dohme Corp.	125	612	20%
National Cancer Institute (NCI)	194	558	35%
Novartis Pharmaceuticals	201	534	38%
Pfizer	62	471	13%

**Table 3.** Top five sponsors with the greatest proportion of missing trials. TrialsTracker, 20/10/2016.

Name of trial sponsor	Trials missing results	Total eligible trials	Percent missing
Ranbaxy Laboratories Limited	35	35	100%
Nanjing Medical University	32	35	91%
Rambam Health Care Campus	27	30	90%
Isfahan University of Medical Sciences	44	49	90%
City of Hope Medical Center	39	44	89%

**Table 4.** Top five sponsors with lowest proportion of missing trials. TrialsTracker, 20/10/2016.

Name of trial sponsor	Trials missing results	Total eligible trials	Percent missing
Shire	0	96	0%
Colgate Palmolive	1	32	3%
Bristol-Myers Squibb	5	115	4%
Eli Lilly and Company	15	292	5%
Johnson & Johnson Pharmaceutical Research & Development, L.L.C.	3	58	5%

### Checks for consistency with previous work

A previous paper automatically matching registry entries to PubMed records and clinicaltrials.gov results found 55% had no evidence of results ^11^, consistent with our overall findings. A previous manual audit (of which BG is co-author) found 56% of trials conducted in the University of Oxford reported results; our method also found 56% for the same institution ^9^. A previous manual audit examined 4347 trials across 51 academic medical centres ^7^. We compared their individual study data against ours and found that 2562 trials (62.6%) in their cohort were also in ours, but note that their study only represented 2% of our total cohort. For studies in both cohorts we found 60% reported results, while they found 66%. Of studies in both cohorts: 1149 were found “reported” by both; 534 studies were found “unreported” by both; 497 were found “reported” by their method and “unreported” by ours; 382 were found “unreported” by theirs and “reported” by ours.

## Discussion

The tool was successfully built, and is now fully functional online. We found non-publication rates consistent with those from previous work using manual searches, and reasonable consistency with individual study matches from a previous manual cohort. A wide range of publication failure rates were apparent in the data.

### Strengths and weaknesses

Our tool is the first to provide live ongoing interactive monitoring of failure to publish the results of clinical trials. The method of automatic matching has strengths and weaknesses. It can be run automatically, at a lower unit cost than a manual search, and therefore allows coverage of more trials than any traditional cohort study. It also permits repeated re-analysis at minimal additional marginal cost compared to a manual search.

In corollary, the efficiency of automatic matching also brings challenges around specificity and sensitivity. Firstly, there may be false adjudications of non-publication, i.e. if a trial’s results paper does not include its registry identifier. However, since 2005 all major medical journals (through the International Committee of Medical Journal Editors; http://icmje.org/recommendations/browse/publishing-and-editorial-issues/clinical-trial-registration.html) have required trials to be registered, and all trials should include their registry ID in the text. Therefore, in our view, the responsibility for results being undiscoverable, when the registry ID is not included by the trialists, lies solely with the trialists; research that is hard to discover is not transparently reported. We hope that in the future better methods for probabilistic record linkage will also be available for wider use ^13^. Secondly, there may be false positives, where a study identified through ID matching and then filtered, is in fact not reporting results. We have used standard filters to account for this, and we are keen to improve our method in the light of concrete constructive feedback. Our checks for consistency against overall prevalence findings and individual study data from previous research to a large extent exclude gross errors in prevalence figures.

Notably there are specific additional methods for linking clinicaltrials.gov records to PubMed records that we tried and rejected. Some trials have a link to a PubMed record directly in the clinicaltrials.gov results_reference tag, which ClinicalTrials documentation ( https://prsinfo.clinicaltrials.gov/definitions.html) suggests indicates results from a publication. We found 2263 eligible trials had such tags, but no summary results on ClinicalTrials.gov. However, on manual examination, we found these are often erroneous, and commonly report results of unrelated studies from several years previously. In discussion, clinicaltrials.gov staff confirmed that this field is neither policed nor subject to substantial editorial control (personal communication with Annice Bergeris).

### Context of other findings

Our findings are consistent with previous work on publication bias ^1^, finding that approximately half of trials fail to report results. Previous studies have used 2007 as their start date for expecting results to be made available, reflecting the FDA Amendment Act 2007. We did not use this date, as this legislation has been widely ignored ^5, 6^, and because we regard sharing results as an ethical obligation, not a legal one. Our methods accept results posting at any time after study completion, and any sponsor posting results for any trial since 2006 will find their results improve in our live data.

### Policy implications

We have previously argued that live ongoing monitoring of trials transparency will help to drive up standards, especially if this information is used by clinicians, policymakers, ethics committees, regulators, patients, patient groups, healthcare payers, and research funders, to impose negative consequences on those who engage in the unethical practice of withholding trial results from doctors, researchers, and patients ^14^. Recent comments by US Vice President Joe Biden threatened to withhold financial support from publicly-funded researchers who fail to report clinical trial results, suggesting some consequences may arise ^6^. We would be happy to collaborate or work with organisations seeking to get a better understanding of their own failure to publish, and wishing to act on this data.

We have also previously argued that medicine has an “information architecture” problem; all publicly accessible documents and data on all clinical trials should be aggregated and indexed for comparison and gap identification, and that good knowledge management and better use of trial identifiers will facilitate this ^15^. At present, medicine faces serious shortcomings in this area. With 75 trials and 11 systematic reviews being published every day on average ^16^ better knowledge management must be a priority.

### Future research

We have shared all our underlying data so that others can explore in detail non-publication for specific studies, interventions, companies, funders, sponsors, or institutions that interest them. We believe that research work on research methods and reporting should go beyond identifying the overall prevalence of problems, and identify individual people and organisations who are performing poorly, in order to both support and incentivise them to improve. That is only possible with ongoing monitoring and feedback on individual studies, an approach we have taken on other projects such as COMPare ^17, 18^. We hope that others will also pursue this model of audit and feedback, and assess its impact on performance.

## Conclusions

We have designed, built, and launched an easily accessible online service that identifies sponsors who have failed in their duty to make results of clinical trials available.

## Software availability

Website available at: https://trialstracker.ebmdatalab.net

Latest source code: https://github.com/ebmdatalab/trialstracker

Archived source code as at the time of publication: DOI: 10.5281/zenodo.163522 ^12^

License: MIT license
