# Supplementary material for: The TrialsTracker: Automated ongoing monitoring of failure to share clinical trial results by all major companies and research institutions
Source: F1000Res. 2016 Nov 3;5:2629. [Version 1] doi: 10.12688/f1000research.10010.1 (PMC5200941; doi:10.12688/f1000research.10010.1)
Supplement: Peer review history

# Peer review: Referee response for version 1

**DOI:** 10.5256/f1000research.10786.r17404

**Published:** 2016-12-19

**Authors:** James Hetherington, Sinan Shi, Jonathan Cooper

**Affiliations:** 1 Research Software Development Group, University College London, London, UK; 2 University College London, London, UK

The authors published an online ranking system which illustrates how the major sponsors share their clinical trial information, in particular through reporting on completed trials. This research offers a new way to automatically identify and match trials registered on ClinicalTrials.gov with their published results in both the ClinicalTrials.gov trial registry and abstracts or metadata of publications (indexed in Pubmed). This automated process can result in a much more frequent update and provide more precise information to the public, in part by encouraging more accessible reporting.

 In this review, we would like to focus our comments on the author’s data processing and software. The authors have provided a code repository containing their website along with some Python code related to the data analysis process. The latter comprises a clear and straightforward IPython notebook detailing all the data analysis steps, including raw data processing, missing trial identification, and validation against other studies. We found it is an intuitive way to present works of this scale, although as discussed later we would like to suggest more modularization. In general, the code is understandable and easy to read. Both unit tests and behavioural tests are included to give more confidence in its reliability. We were able to re-run the entire IPython notebook with only some minor modifications.

 We do have some minor comments and suggestions regarding the coding quality and reproducibility aspects of this project.

- We have noticed that the XML parsing and Pubmed data extraction parts break easily due to variations in the source files or network problems. It would therefore be beneficial to make these two parts into functions with associated unit tests to ensure the correctness and robustness of the code.
- Compounding the problem, these parts also take a very long time to compute. We left the program running for several days trying to update the trial-abstract database, only to have it fail part-way through. Further incremental updating mechanisms would help greatly here, for instance adding an extra column to the database to register the last search date so that recently searched entries will not be queried again.
- One hopes that the ‘live’ website is updated from time to time with more recent results. It would be nice to have details on how frequently this happens - is it an automated process?
- The current data on Github have some small differences compared to the results presented in the paper. We can fully understand that the data in the repository should be updated, and the development is an ongoing process. However, it would have been good from an audibility point of view to make the data which have been used for the paper available. For instance, the specific git commit id used for the paper could be given in the paper itself and the repository’s README.
- A requirements.txt is provided in the source code to facilitate installing the project’s dependencies, however, not all of the dependencies are on the list. Changes in recent versions of some of these cause the code to break. Please specify all the dependencies (even indirect ones) including the versions used in the requirements.txt file. We have submitted a pull request with the list we found worked.

Overall, the new tool offered by the authors enables more frequent and larger-scale identification of whether trials have been reported. Their code is clear and reflects the methodology faithfully. This tool will help in the push for improving clinical trial transparency.

We have read this submission. We believe that we have an appropriate level of expertise to confirm that it is of an acceptable scientific standard.

# Peer review: Referee response for version 1

**DOI:** 10.5256/f1000research.10786.r17811

**Published:** 2016-11-21

**Authors:** Andrew P. Prayle

**Affiliations:** 1 Division of Child Health, University of Nottingham, Nottingham, UK

**Title and abstract**

 The title is appropriate and discusses the content of the paper in one sentence.  The abstract starts generally, drills down into the methods concisely and discusses the contribution to the literature which this manuscript and software project appropriately. 

**Article content**

 Powell-Smyth and Goldacre report on a piece of work which will make a substantial contribution to  the clinical trials enterprise. 

 They have developed an open source web application which automatically takes data from the US based ClinicalTrials.gov registry and searches for results (either summary results on ClinicalTrials.gov or an abstract on PubMed).  The software then ranks study sponsors by the proportion of trials which have reported results. 

 This approach is novel in its approach to on-line availability of data. This means that the dataset is easily searchable through a web based application.  Automated systems have been explored in the past (e.g. Huse *r et al* 2013), as have manual searches (Tompson 2016), and the results of the automated system presented appear consistent with these. 

 I have reviewed the online web based software, this is simple to use and demonstrates the ability of the approach to hold institutions which sponsor research to account, by summarising their contribution of results to the clinical trial literature.

 The central contribution is an automated system for determining if a trial registered on ClinicalTrials.gov has published summary records on clinicaltrials.gov, or has an abstract indexed on PubMed.  The work hinges on whether their automated system can in fact do this. The authors make a persuasive case that they are able to find summary results and abstracts where these have been published.  They provide what they have said they can do in the on-line Jupyter notebook.  Additionally, the open source code in the Github repository is straightforward to read, and supports their case.  Finally, I downloaded the full dataset and explored it, and in the cases which I looked at their spreadsheet had correctly identified completed trials and the accompanying Pubmed abstract. 

 Therefore, although there may be a few trials which have been misclassified, I think that the methods used appear very robust.  Additionally, if trials have been misclassified, the authors give suggestions of how to adjust this through changes to the journal entries on Pubmed, or through summary results on Pubmed. 

 In the discussion the strengths and limitations of their automated approach are carefully elaborated upon.  The key strength is that a large proportion of the clinical trials landscape is included in their study.  The limitation is of course that automated analysis may incorrectly label some trials as unreported when in fact they are, but my assessment of their raw data is that this must be infrequent as I have not been able (in and admittedly unscientific sample obtained by scrolling through the raw data, and looking at trials which I am familiar with if I see them) to identify such a case. 

**Conclusions**

 The authors state that they present this work to aim to improve the clinical trials landscape in terms of the ‘information architecture’ of missing results. I believe that we should take this work at face value as a genuine, innovative approach which is trying to improve the problem of non-reporting, by giving transparency of reporting at the study sponsor level.  It is reported carefully. The data presented back up the case for a clear need for improved trial reporting. 

**Data**

 This study is an exemplar of how to publish reproducible research.  The data and code and extensive documentation are available and free to download and explore.  My only suggestion is to have a second repository in case GitHub disappears.

I have read this submission. I believe that I have an appropriate level of expertise to confirm that it is of an acceptable scientific standard.
